# Regulation of Angiotensin-Converting Enzyme 2: A Potential Target to Prevent COVID-19?

**DOI:** 10.3389/fendo.2021.725967

**Published:** 2021-10-22

**Authors:** Yue Hu, Lihuan Liu, Xifeng Lu

**Affiliations:** Department of Physiology, Basic Medical College, Shenzhen University, Shenzhen, China

**Keywords:** SARS-CoV-2, COVID-19, angiotensin-converting enzyme 2, renin–angiotensin system, therapeutic target

## Abstract

The renin–angiotensin system (RAS) is crucially involved in the physiology and pathology of all organs in mammals. Angiotensin-converting enzyme 2 (ACE2), which is a homolog of ACE, acts as a negative regulator in the homeostasis of RAS. ACE2 has been proven to be the receptor of severe acute respiratory syndrome coronavirus 2 (SARS-CoV-2), which caused the coronavirus disease 2019 (COVID-19) pandemic. As SARS-CoV-2 enters the host cells through binding of viral spike protein with ACE2 in humans, the distribution and expression level of ACE2 may be critical for SARS-CoV-2 infection. Growing evidence shows the implication of ACE2 in pathological progression in tissue injury and several chronic conditions such as hypertension, diabetes, and cardiovascular disease; this suggests that ACE2 is essential in the progression and clinical prognosis of COVID-19 as well. Therefore, we summarized the expression and activity of ACE2 under various conditions and regulators. We further discussed its potential implication in susceptibility to COVID-19 and its potential for being a therapeutic target in COVID-19.

## Introduction

Coronavirus disease 2019 (COVID-19) is an acute respiratory disease caused by severe acute respiratory syndrome coronavirus 2 (SARS-CoV-2) (1). It was first reported in December 2019 in Wuhan, Hubei Province of China; as of March 24, 2021, it has resulted in more than 100 million infections and 3.48 million deaths worldwide. SARS-CoV-2 is predominantly transmitted through direct contact or respiratory droplets in a proximity- and time-dependent manner, and it often requires close contact of within 6 feet over a period of 15 min or longer (2). Common COVID-19 symptoms include fever, dry cough, fatigue, and dyspnea, whereas severe symptoms are accompanied by systemic infection and pneumonia because the lungs are the primary target of the disease (3). The disease also causes damages to other organs such as the heart, liver, and kidneys as well as organ systems such as the intestinal, circulatory, and immune systems. Infected patients experience different symptoms to different degrees, ranging from asymptomatic, mild respiratory infections to severe acute respiratory syndrome, which results in organ failure, shock, acute respiratory distress symptoms, heart failure, arrhythmias, renal failure, and eventually death (4).

Angiotensin-converting enzyme 2 (ACE2) has been reported to be a potent negative regulator of the renin–angiotensin system (RAS), which encodes protein as a functional receptor for the spike glycoprotein of the human coronaviruses SARS, SARS-CoV-2, and HCoV-NL63, and it is a key pathogenic factor for coronavirus infection in host cells (5, 6). In the RAS, ACE2 degrades angiotensin (Ang) II and converts it into Ang-(1-7), which is a vasodilative, antiproliferative, and antiapoptotic agent (7). Thus, the balance between Ang I/Ang II and Ang-(1-7)/Ang-(1-9) in the RAS is disrupted by the combination of coronaviral spike glycoprotein and ACE2 (8), which changes the permeability of cell membranes and leads to organ damage. In particular, SARS-CoV-2 infects type II alveolar epithelial cells through ACE2, thus inducing lung injury (9). The virus then continues to invade the cells of other organs such as the heart, kidney, liver, and intestine by binding the ACE2 receptor through blood circulation; this triggers an excessive immune response, which produces numerous inflammatory cytokines and an imbalance in T-helper-1 and T-helper-2 cells, thus causing a cytokine storm and ultimately leading to multiple organ dysfunction syndrome (10).

As mentioned above, the entry of SARS-CoV-2 into host cells is facilitated through the binding of the viral spike protein with the extracellular domains of the transmembrane ACE2 proteins, resulting in the downregulation of surface ACE2 expression (11). Clinical research has indicated a direct link between the downregulation of tissue ACE2 and the imbalance of the RAS in patients with COVID-19, which promotes the development of multiorgan injuries caused by SARS-CoV-2 infection (12). Because of the crucial role of ACE2 in SARS-CoV-2 infection, potential therapeutic strategies include the prevention of the binding of human ACE2 and the receptor-binding domain of the viral spike protein or the direct and indirect regulation of ACE2 expression, small molecule inhibitors, drugs, ACE2 antibodies, or single-chain antibody fragments against ACE2, which may influence its activity. In addition, a novel strategy for the rapid detection of SARS-CoV-2 has been reported (13), which is based on the function of ACE2 as a receptor of the spike protein and detects samples in a lateral flow immunoassay without DNA extraction and quantitative reverse transcriptase-polymerase chain reaction.

Therefore, understanding the expression and activity of ACE2 regulation in various conditions may help predict SARS-CoV-2 infection in patients with COVID-19 under different conditions and clinical prognosis. In this review, we summarize the regulation of ACE2 expression and activity under various conditions and regulators and discuss its role as a potential therapeutic target in COVID-19.

## RAS and ACE

**Figure 1:**
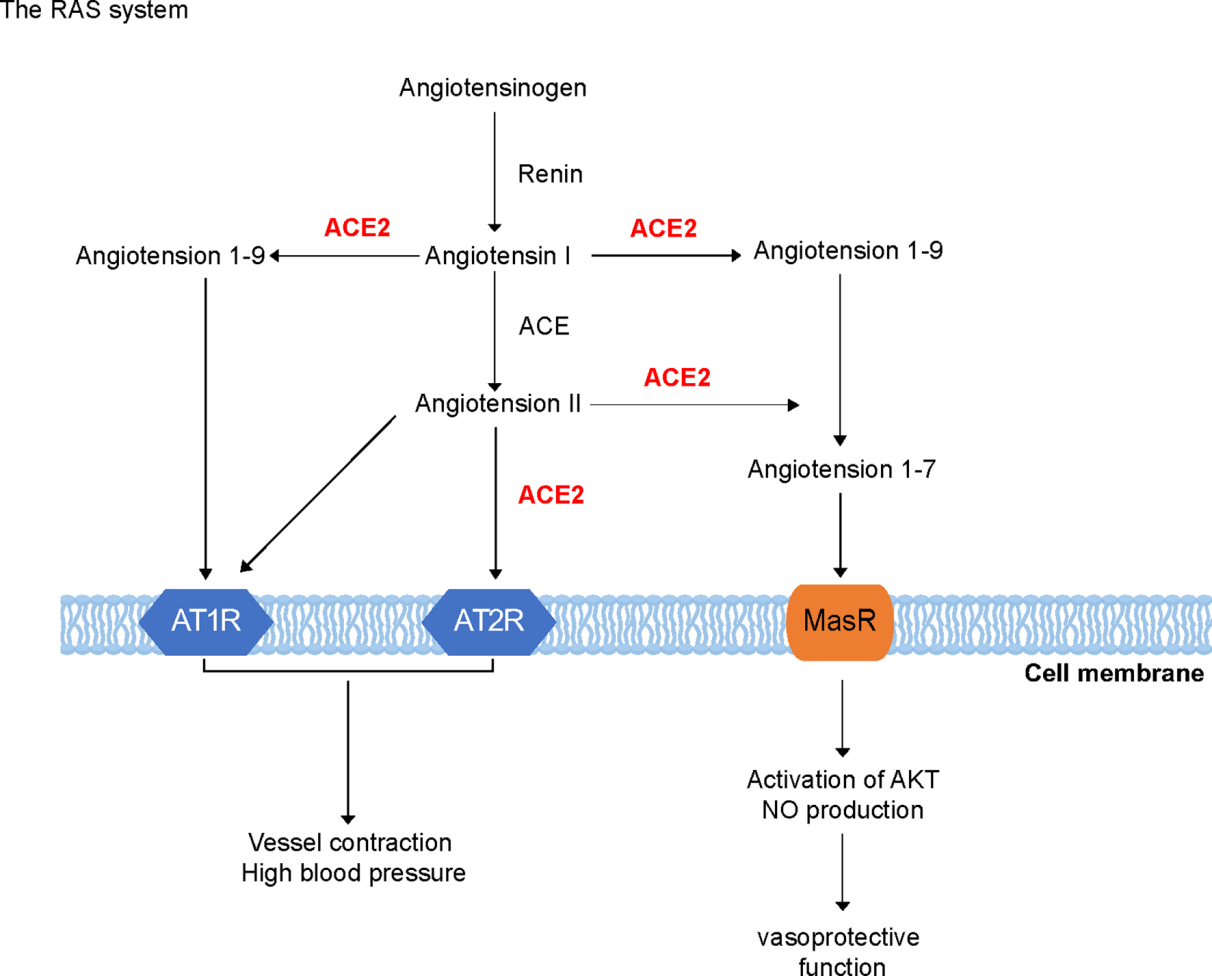
Renin–angiotensin system. Renin cleaves angiotensinogen to form Ang I, which is then cleaved by angiotensin-converting enzyme (ACE) to generate Ang II. Ang II can bind to Ang II type 1 and 2 receptors (AT1/2R). ACE2 is a single transmembrane protein, which can recognize Ang I and cleave it into Ang 1-9, which is quickly converted to Ang 1-7 by ACE. Moreover, Ang-(1-7) has been identified as a ligand for Mas receptor, which is a seven-transmembrane G-protein coupled receptor. Mas receptor may induce intracellular signaling cascades such as protein kinase B and induction of nitric oxide (NO) production.

The RAS is a major regulator of blood pressure and fluid homeostasis (**Figure 1**). It interacts with the kallikrein–kinin system and plays a key role in the cardiovascular system (14). Renin cleaves angiotensinogen to form Ang I, which is then cleaved by ACE to generate Ang II. Ang II can bind to Ang II type 1 and 2 receptors (AT1/2R) and induce vessel contraction and increase blood pressure, thus acting as an effector of the RAS (15, 16). A homolog of ACE, ACE2, was discovered approximately two decades ago (17). This homolog is a single transmembrane protein with 805 amino acids, containing a HEXXH zinc-binding domain that is homologous to the enzyme activity site of ACE, sharing 42% similarities with ACE at the amino acid level (18). However, ACE2 is insensitive to classic ACE inhibitors (ACE-Is) such as captopril, and it plays a role completely opposite to that of ACE (19). ACE2 can recognize Ang I and cleave it into Ang-(1-9), which is quickly converted to Ang-(1-7) by ACE (19, 20). Moreover, ACE2 isolated from the human heart only hydrolyzes Ang II but not Ang I. These findings suggest that Ang II is the preferred substrate of ACE2 (21). Thus, ACE2 can act as an antagonist of ACE functions by degrading Ang II and its consequent vasoconstrictive effects. Furthermore, Ang-(1-7) has been identified as a ligand for Mas receptor, which is a seven-transmembrane G-protein-coupled receptor. Upon ligand binding, Mas receptor induces intracellular signaling cascades, including the activation of protein kinase B and induction of nitric oxide production, exerting vasoprotective functions in contrast to the hypertensive and proliferative functions of Ang II–AT1R/AT2R axis (22). Moreover, ACE2 can act on not only the RAS but also several peptides from other systems, such as neurotensin (1–14), apelin13, dynorphin (1–14), and some of the kinin metabolites (23, 24).

Unlike the ubiquitous expression of ACE, ACE2 is predominantly expressed in the heart, kidneys, and testes. However, ACE2 expression can also be observed in the gastrointestinal tract, lungs, and liver but to a lesser extent. In general, genetic deletions of ACE2 impair cardiovascular functions, but the exact impacts are various. Crackower et al. (25) found that ACE2 deficiency severely reduces cardiac contractility but it does not affect blood pressure, whereas Gurley et al. (26) reported that ACE2 knockout (KO) mice showed no changes in the cardiac structure or function; however, they showed slight increases in blood pressure. Moreover, ACE2 KO mice were shown to be more susceptible to Ang II-induced hypertension (26). In addition to its effects on the cardiovascular system, ACE2 deficiency influences the functions of the liver, whereas its expression is relatively abundant. In addition, ACE2 KO mice have been reported to show abnormal lipid accumulation in the liver and impaired glucose metabolism (27). Furthermore, ACE2 plays a protective role against liver damage; the loss of ACE2 function in the liver may accelerate the development of liver injury such as liver fibrosis (28). In the lungs, ACE2 is primarily expressed in epithelial cells. Deleting ACE2 does not impair lung functions but augments lung injury induced by cigarette smoke exposure, which is likely to be due to the increased activation of matrix metalloproteinases.

**Figure 2:**
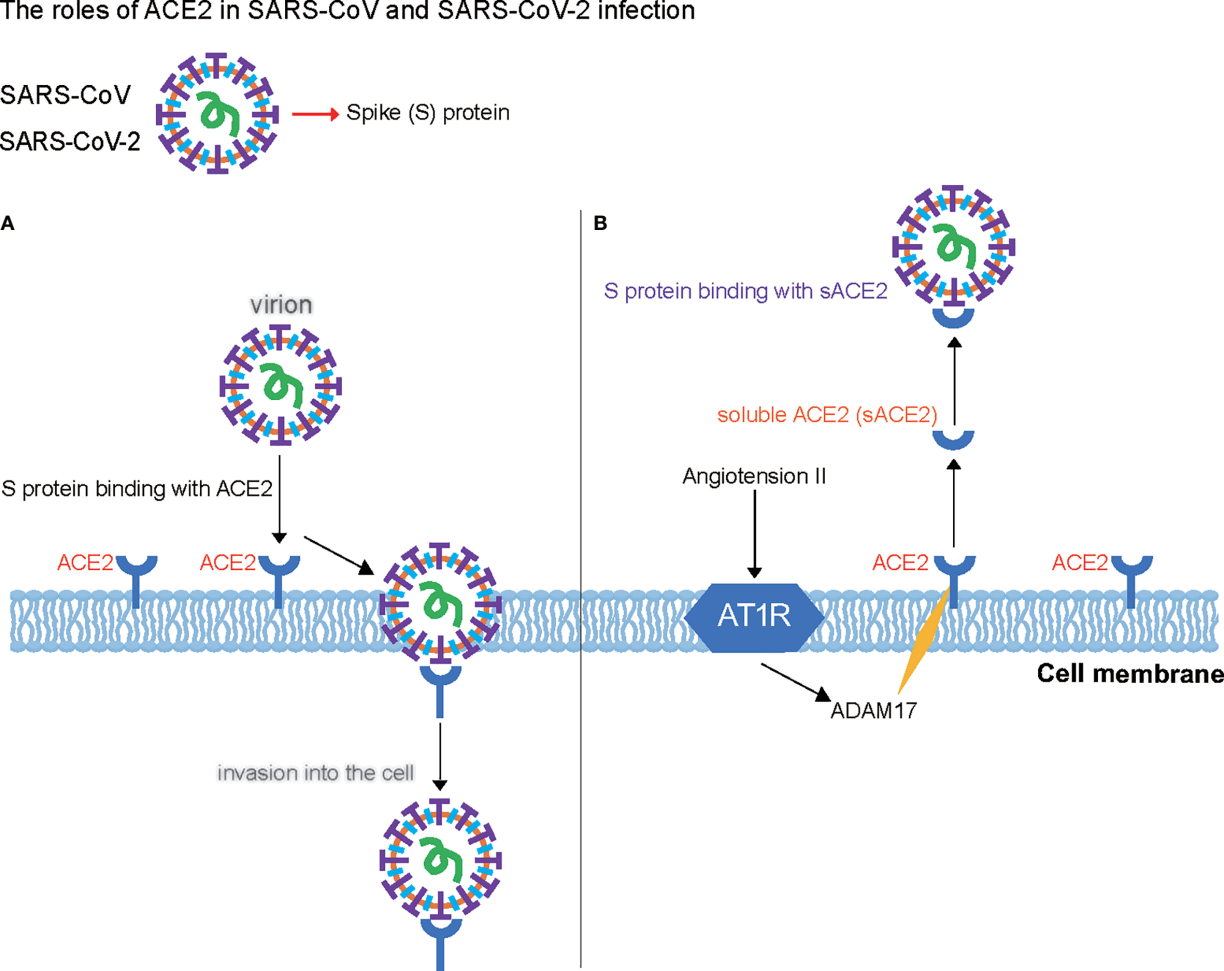
Roles of angiotensin-converting enzyme 2 (ACE2) in severe acute respiratory syndrome coronavirus (SARS-CoV) and SARS-CoV-2 infection. **(A)** ACE2 acts as the key receptor in avian influenza H5N1 and H7N9 virus infections as well as in SARS-CoV and SARS-CoV-2 infections. **(B)** Roles of a soluble form of angiotensin-converting enzyme 2 (sACE2) and A disintegrin and metalloprotease 17 (ADAM17) in cells infected with SARS-CoV and SARS-CoV-2. ADAM17 cleaves ACE2 and forms a 20-amino acid transmembrane peptide. The binding of Ang II with AT1R promotes sACE2 formation by increasing ADAM17 activity, which generates sACE2 and binds to the spike protein of SARS-CoV-2 as the virus receptor.

ACE2 plays a crucial role in avian influenza H5N1 and H7N9 virus infections as well as in SARS-CoV and SARS-CoV-2 infections (**Figure 2A**). Furthermore, ACE2 deficiency increases the severity of H7N9-virus-induced lung injury, which may be attenuated by blocking AT1R, suggesting that enhanced Ang II/ATR1 signaling is a major cause of lung injury during infection with the avian influenza virus. Notably, although ACE2 receptor mediates virus infection, both SARS-CoV and H7N9 virus downregulate its expression in the lungs shortly after viral infection; however, the expression of ACE remains unaffected. Thus, it is not surprising that serum Ang II levels were increased in patients with H5N1 and H7N9 infection. Moreover, the administration of human recombinant ACE2 protein reduces lung injury induced by the avian influenza H5N1 virus (29, 30).

## Soluble ACE2 and Recombinant ACE2

In addition to membrane-bound ACE2, a soluble form of ACE2 (sACE2) also exists in circulation. ACE2 may be constitutively cleaved to release two distinct major soluble forms. The deglycosylated molecular masses of the larger and smaller soluble forms are approximately 80 and 70 kDa, respectively (31). A 70-kDa ACE2 was purified from astrocyte cell culture, which converted Ang II into Ang-(1-7), suggesting that this protein is a secreted form of the enzyme (32). A 60-kDa sACE2 was also purified from the mesangial cells from mice, which generated Ang-(1-7) from Ang II that prevented exposures to high levels of this vasoconstrictive peptide and exert a protective effect in renal hemodynamics (33). Because sACE2 contains a completely catalytic domain, it may still be capable of cleaving Ang II in circulation. Circulating sACE2 activity levels have been associated with chronic systolic heart failure in humans, and after intensive medical therapy, increases in baseline serum sACE2 levels have predicted a significant reduction in the risk of death after cardiac transplantation (34).

sACE2 is enzymatically active and partially inhibits virus entry into the target cells of human airway epithelia. Mutant and chimeric ACE2 proteins showed that a point mutation in the ACE2 ectodomain, L584A, markedly attenuated shedding. The resultant ACE2-L584A mutant trafficked to the cell membrane and facilitated SARS-CoV entry into target cells, suggesting that the ACE2 ectodomain regulates its release and that residue L584 might be part of a putative sheddase “recognition motif”. Both wild-type ACE2 and the ACE2-L584A mutant supported productive infection with the SARS-CoV, indicating that sACE2 generation is not required for the protein to function as a coronavirus receptor (35). Soluble recombinant ACE2 (rACE2) has been reported to prevent the rapid hypertension elicited by Ang II by reducing its level and increasing Ang-(1-7) level in plasma, and during Ang II infusion, rACE2 degraded Ang II and thus normalized blood pressure (36). Thus, rACE2 may provide a novel therapeutic target and be used as a potential antihypertensive drug. Notably, ADAM17, which is a disintegrin and metalloprotease 17, cleaves ACE2 between Arg (708) and Ser (709), forming a 20-amino acid transmembrane peptide (37). The binding of Ang II with AT1R can promote sACE2 formation by increasing ADAM17 activity (38), which generates sACE2, reducing surface ACE2 expression (**Figure 2B**). We suggest that rACE2 can be important in SARS-CoV-2 treatment.

Overall, on the basis of the known molecular functions of the RAS, ACE2 and related genes play central roles in the RAS activity, associated pathologies, and virus infection, including the global pandemic, COVID-19. Thus, it is extremely necessary and useful to summarize the regulators of ACE2 for further research.

## Transcriptional and Translational Regulation of ACE2

### Transcription Factor Regulation

Similar to other components of the RAS, ACE2 expression is also well regulated to maintain appropriate RAS activities. Ang II can suppress ACE2 expression *via* AT1R in neuronal cells, forming a negative feedback loop. Ang-(1-7) can antagonize the Ang II-induced downregulation of ACE2 expression, although it does not influence ACE2 expression in neuronal cells. It has been revealed that the regulation of ACE2 by Ang II/AT1R requires sequences to lie between 481 and 516 base pairs upstream of the transcription initiation site of human ACE2; although the exact transcription factor (TF) mediating Ang II/AT1R-regulated ACE2 expression is yet to be identified, it is evident that the ATTTGGA motif, which is a binding sequence for TF Ikaros, is indispensable for this regulation (39). There are also three binding sites that are highly conserved in mammals for hepatic nuclear factor 1 (HNF1), which is a TF that regulates various hepatic genes and plays an important role in homeobox gene family expression of the liver and kidney. Knocking out HNF1 in mice induces death around weaning after a progressive wasting syndrome with a markedly enlarged liver (40). Both HNF1α and HNF1β can increase ACE2 expression in insulin-producing cells, including pancreatic β cells and insulinoma cells. Notably, HNF1α increases ACE2 gene expression in primary cells from pancreatic islets through evolutionarily conserved motifs in the proximal promoter region (41). In addition, HNF4α—a master regulatory protein in the liver and an important TF in angiotensinogen gene regulation—targets ACE2 and influences its mRNA expression (42).

Hypoxia-inducible factor 1 (HIF-1) is a heterodimeric TF comprising a regulatory α subunit (HIF-1α) and a constitutive β-subunit (HIF-1β) (43). Notably, HIF-1α indirectly regulates ACE2 through the downregulation of ACE2 expression for the accumulation of Ang II catalyzed by ACE. The expression levels of ACE2 mRNA increase during the early stages of hypoxia and decrease to near baseline levels at the later stages after HIF-1α accumulation in pulmonary artery smooth muscle cells. Therefore, direct regulation of ACE and bidirectional regulation of ACE2 by HIF-1α during hypoxia could play a protective role during the development of hypoxic pulmonary hypertension (44).

### Epigenetic Regulation

Except for the TF, epigenetic regulation could also occur in ACE2 mRNA expression, such as the regulation of histone acetylase/deacetylase and microRNAs. Silent information regulator T1 is a histone deacetylase and a transcriptional mediator, which exerts protective effects *via* the deacetylation of its target proteins such as proteins involved in cellular stress resistance and genomic stability. Silent information regulator T1 binds to the ACE2 promoter; this binding may increase after treatment with the antimicrobial peptide mimic 5-aminoimidazole-4-carboxamide ribonucleotide (AICAR) in Huh7 cells, which increases ACE2 mRNA expression levels under the hypoxic conditions induced. In contrast, the inhibition of Silent information regulator T1 activity eliminates the 5-aminoimidazole-4-carboxamide ribonucleotide-induced increase in ACE2 expression (45). The ACE2 mRNA expression level increases by increasing the occupancy of histone H3 acetylation, which binds on ACE2 promoter region in rabbit models of high-cholesterol diet-induced atherosclerosis treated with atorvastatin. Additionally, the epigenetic regulation of ACE2 may be another realistic way to treat atherosclerosis and cardiovascular disorders (46).

MicroRNAs are endogenous, small (19–25 nucleotides), and non-coding RNAs, which can target specific genes and function as the negative regulators of gene expression by inhibiting the translation of or promoting the degradation of targeted mRNAs. The role of microRNAs in regulating the standard and novel cardiac RAS during aerobic exercise training in rats with left ventricular hypertrophy has indicated that exercise can increase miRNA-27a and miRNA-27b targeting ACE and miR-143 targeting ACE2, inducing higher mRNA expression and protein levels of ACE2, followed by an increase in Ang-(1-7) and AT2R (47) levels. Additionally, in patients with uremia, circulating miR-421 shows enhanced higher expression than that in healthy individuals; it targets leucocytic ACE2 and decreases its transcripts. The association between miR-421 and ACE2 may contribute to lower expression of the enzyme in the leukocytes of chronic kidney diseases, further supporting the development of atherosclerotic events (48), which suggests that miRNA can also be a target for atherosclerosis. In cardiovascular disease, miR-421 could be a potential regulator of ACE2 involved in the development of thrombosis and downregulation of its protein level in both primary cardiac myofibroblasts and transformed cells. Notably, miR-421 levels show significant interpatient variabilities, which are consistent with the alteration in ACE2 expression (49).

### Regulation by Other Factors

Several factors regulate ACE2 mRNA expression levels during disease progression. For example, transforming growth factor-β, which is pivotal in diabetic nephropathy, has a negative feedback effect on the mRNA expression and protein level of ACE2 and its cell membrane-binding receptor Mas. Thus, it can decrease ACE2, Mas, and Ang-(1-7) conversion from Ang II in high glucose-cultured NRK-52E cells, suggesting a possible treatment for diabetic renal fibrosis (50). Additionally, nuclear factor erythroid 2-related factor 2 functions as a master regulator of redox balance in cellular cytoprotective responses. The loss of this factor upregulates the expression of renal proximal tubule cellular ACE2 and its Mas receptor, followed by an increase in urinary Ang-(1-7) levels and downregulation of the expression of angiotensinogen, ACE, and profibrotic genes in Akita mice (51).

Curcumin, a yellow pigment extracted from the rhizomes of *Curcuma longa*, which exhibits pharmacologic properties such as anti-inflammation and fibrosis properties, increases ACE2 protein levels and enhances its expression in the intermyocardium relative to animals with Ang II infusion (52). Rosiglitazone, which is a peroxisome proliferator-activated receptor gamma ligand that acts as an insulin sensitizer and exerts cardiovascular action, increases ACE2 and Ang-(1-7) protein levels and decreases the Ang II level to lower blood pressure in a peroxisome proliferator-activated receptor gamma-dependent manner in male Wistar rat models (53).

### Regulation by Chinese Medicine

As mentioned above, ACE2 is an important enzyme that attaches to the cellular membranes in the lungs, arteries, heart, kidney, and intestines and functions in the pathophysiology of lung and cardiovascular diseases. Some drugs regulate ACE2 protein levels during disease processes. For example, Yinhenhao decoction is a traditional Chinese medicine that has antifibrotic effects that reduce hepatic fibrogenesis in the bile duct ligation rat liver model. This is achieved by decreasing the standard RAS pathway components and transforming growth factor-β1 down expression, which elevates the ACE2 protein level to recover and rebuild self-regulation of the RAS (54). In addition, Sini decoction, which is used widely for treating clinical diseases, alleviates *Escherichia coli*-induced acute lung injury in mice by markedly enhancing ACE2 protein levels to activate the ACE2–Ang-(1-7)–Mas pathway and equilibrate the ACE–Ang II–AT1R and ACE2–Ang-(1-7)–Mas axis (55). Diminazene aceturate, which is the most widely used therapeutic agent for trypanosomiasis and has been shown to prevent pulmonary hypertension, increases the protein level of ACE2 to prevent the progression of asthma by altering the levels of AKT, p38, NF-κB, and other inflammatory markers in male Wistar rats (56). Some other regulators such as RAS regulators and drugs influence ACE2 functions at transcriptional and translational levels and its activities.

On the basis of these ACE2 regulators described above, we summarized the available data, including the data regarding their effects on ACE2 through mRNA expression, protein level alteration, and activities in **Table 1**.

**Table 1:** Transcriptional and translational regulators of angiotensin-converting enzyme 2.

Class	Gene name	Condition	System	Impact on ACE2
Transcription factor	Hepatic nuclear factor 1 (HNF1), comprising HNF1α and HNF1β	Healthy	*In vivo* in insulin-producing cells, including pancreatic β cells and insulinoma cells	Increase ACE2 mRNA expression level (40, 41)
	HNF4α	Healthy	*In vivo* in the liver of mice	Target ACE2 and affect its mRNA expression level (42)
	Hypoxia-inducible factor 1 (HIF-1), comprising HIF-1α and HIF-1β	Hypoxic pulmonary hypertension	*In vivo* in pulmonary artery smooth muscle cells	Target ACE2 and indirectly decrease ACE2 mRNA expression level for the accumulation of Ang II catalyzed by ACE (43, 44)
Epigenetic regulation	Histone deacetylase: silent information regulator T1	Hypoxic	*In vivo* in Huh7 cells	Bound to the ACE2 promoter and increase ACE2 mRNA expression level (45)
	Histone H3 acetylation	Atherosclerosis	In the heart of rabbits with high-cholesterol diet-induced atherosclerosis treated with atorvastatin	Bound to the ACE2 promoter and increase ACE2 mRNA expression level (46)
	MicroRNAs: miR-143	Left ventricular hypertrophy	In aerobic exercise training rats	Increase ACE2 mRNA expression and protein level (47)
	MicroRNAs: miR-421	Uremic	Human	Target leucocytic ACE2 and decrease its transcripts (48)
		Thrombosis	In both primary cardiac myofibroblasts and transformed cells	Decrease ACE2 protein level (49)
Other factors	Transforming growth factor-β	High-glucose-cultured	In NRK-52E cells	Decrease ACE2 mRNA and protein level (50)
	Nuclear factor erythroid 2–related factor 2 (Nrf2)	Nrf2 knockout	In Akita mice renal proximal tubule cells	Increase ACE2 expression (51)
	Curcumin	Myocardial fibrosis	In male Sprague-Dawley rats	Increase ACE2 protein level (52)
	Rosiglitazone: a peroxisome proliferator-activated receptor gamma ligand	Hypertension	*In vivo* in male Wistar rats	Increase ACE2 protein level (53)
Chinese medicine	Yinhenhao decoction	Hepatic fibrogenesis	*In vivo* in the bile duct ligation rat liver model	Increase ACE2 protein level (54)
	Sini decoction	Acute lung injury	*In vivo* in rats	Increase ACE2 protein level (55)
	Diminazene aceturate	Asthma	*In vivo* in male Wistar rats	Increase ACE2 protein level (56)

## Pharmacological Regulation of ACE2

Considering that the primary symptoms of COVID-19 reported to date include hypertension, atherosclerosis, diarrhea, glaucoma, anosmia, ageusia, skin lesions (dermatitis), autoimmune inflammation of the central nervous system, and damage to organs such as the lungs, heart, kidneys, and testicles, all these diffuse COVID-19 disorders are likely associated with an overreaction of the RAS in patients with COVID-19 (57). Current pharmacotherapies aim to inhibit multiple levels of the RAS through distinct modes of action. Because ACE2 is the key negative modulator of the RAS, its gene transcription and translation level and the catalytic activities are modified owing to the intricate nature of the RAS. The presence of several RAS modulators functioning at different levels in this system results in various effects on ACE2. In **Table 2**, we summarize the major studies, including the effects of various RAS modulators (as drugs) on ACE2 in mRNA expression, protein levels, and activities. The effects elicited by these different drugs on ACE2 depend on several factors, including the various systems studied, disease progression stage, and drug usage.

**Table 2:** Studies investigating the impacts of several cardiopulmonary diseases and renin–angiotensin system modulators on angiotensin-converting enzyme 2 expression and activity.

Drug class	Drugs	Condition	System	Impact on ACE2
ACE-I	Lisinopril	Healthy	*In vivo* in rat renal cells	Decrease in ACE2 mRNA expression (combination with low-sodium diet) (58)
			*In vivo* in rat cardiac (LV) cells	Increase in ACE2 mRNA expression (59)
		HTN	*In vivo* in transgenic Ren2 rats cardiac and renal cells	Increase in ACE2 mRNA expression and activity (60)
	Enalapril	MI	*In vivo* in rat cardiac (LV) and plasma cells	Increase in ACE2 mRNA expression and activity 8 weeks post-MI (61)
	Captopril	ALI	*In vivo* in rat pulmonary tissue and *in vitro* in rat pulmonary microvascular endothelial cells	Increase in ACE2 protein level (62)
	Any ACE inhibitors	Likely HTN	*In vivo* in human intestinal cells	Increase in intestinal ACE2 mRNA expression (63)
ARB	Losartan	Healthy	*In vivo* in rat cardiac (LV) cells	Increase in ACE2 mRNA expression and activity (64)
			*In vivo* in rat cardiac (LV)/renal cells	Potentiation of renal upregulation of ACE2 mRNA expression (65)
		ARDS	*In vivo* in rat BALF	Increase in ACE2 activity (66)
	Olmesartan	HTN	*In vivo* in rat aorta/carotid artery cells	Increase in ACE2 mRNA expression and activity (67)
	Irbesartan	Healthy	*In vivo* in rat aorta cells	Significantly increase mRNA expression and protein level (68)
	Telmisartan	HTN	*In vivo* in rat cardiac (aorta) cells	Decrease in ACE2 activity (69)
		Healthy	*In vivo* in rat renal cells	Increase in ACE2 mRNA expression and protein level (70)
	Eprosartan	HF	*In vivo* in rat cardiac cells	Increase in ACE2 activity (71)
MRB	Eplerenone	Healthy	*In vivo* in rat peritoneal macrophages/cardiac cells	Increase in ACE2 mRNA expression and activity (72)
	Spironolactone	HF	*In vivo* in human monocyte-derived macrophages	Increase in ACE2 mRNA expression and activity (72)
RI	Aliskiren	DN	*In vivo* in rat renal cells	Decrease in ACE2 activity (73)

ACE-I, angiotensin-converting enzyme inhibitor; ALI, acute lung injury; ARB, angiotensin-receptor blocker; DN, diabetic nephropathy; HF, heart failure; HTN, hypertension; LRTI, lower respiratory tract infection; LV, left ventricle; MI, myocardial infarction; MRB, mineralocorticoid receptor blocker; RI, renin inhibitor.

Although ACE2 is not the direct cellular target of these therapies, ACE2 gene transcription, translation, and catalytic activity are also modified owing to the intricate nature of the RAS. For example, angiotensin-receptor blockers (ARBs) and ACE-Is mostly increased ACE2 mRNA expression and protein level in the heart, kidneys, and thoracic aorta, but the activity varies across experimental models and tissues for ACE-Is (**Table 2**). Lisinopril treatment in healthy rats fed with low-sodium diet decreases the ACE2 mRNA levels in renal cells (58), whereas it increases ACE2 mRNA expression in cardiac cells (59). In addition, lisinopril used in transgenic Ren2 rats in hypertension increases ACE2 mRNA level and activity in the heart and kidneys (60). Moreover, in other ACE-I treatments, ACE2 mRNA expression and activity are both increased by enalapril in myocardial infarction rat cardiac and plasma cells (61). ACE2 protein levels are increased by captopril in rat pulmonary tissue and pulmonary microvascular endothelial cells *in vitro* under conditions of acute lung injury (62). Moreover, ACE2 mRNA expression levels were increased in human intestinal cells by any ACE-Is under hypertension condition (63). These findings may be attributed to the tissue-specific regulation of ACE2 as higher ACE2 protein levels were reported in the heart, but ACE2 activity was higher in the kidneys of Sprague–Dawley rats, adding to the complexity of the tissue in the RAS (74). Therefore, the mechanisms behind the augmentation of ACE2 mRNA levels by ACE-Is and ARBs require further characterization. Moreover, mineralocorticoid receptor blockers such as spironolactone, which prevents increases in both ACE and AT1R mRNA levels and the associated increase in AT1R density from aldosterone signaling in cardiomyocytes (75, 76), increase ACE2 mRNA expression and activity in monocyte-derived macrophages from patients with chronic heart failure (72). Furthermore, eplerenone increases ACE2 mRNA expression and activity in healthy rat peritoneal macrophages or cardiac cells (72). The renin inhibitor, aliskiren, decreases the ACE2 activity in diabetic nephropathy renal cells (**Table 2**) (73).

Clinical researchers have reported that patients with COVID-19 with these comorbidities of cardiovascular disease, diabetes, and chronic hypertension have been treated with RAS modulators such as ACE-Is, ARBs, mineralocorticoid receptor blockers, and renin inhibitors (77). ACE-Is and ARBs are commonly used therapeutic drugs for hypertension. Animal experiments have revealed that these drugs decrease the systolic pressure in healthy rats and upregulate ACE2 levels (78). However, some researchers still doubt the safety and effect of using these drugs on patients with COVID-19. As ACE2 expression is suppressed in hypertension and may be further deprived by the SARS-CoV-2 upon infection, the application of ARBs may protect against pulmonary injury under careful blood pressure management.

## Conclusions

Since the discovery of ACE2, progress has been made in elucidating its biochemical actions and fundamental role in cardiovascular diseases and as a receptor for SARS-CoV-2 attachment. ACE2 functions a negative regulator of the RAS by metabolizing Ang II into the beneficial peptide Ang-(1-7); this important biochemical and physiological property is being harnessed as a potential therapeutic target in patients with cardiovascular diseases. The activation of the RAS axis due to the binding of SARS-CoV-2 to ACE2, which leads to the direct loss of ACE2 and indirect loss of ACE2 *via* proteolytic processing and shedding, partly drives the systemic manifestations of COVID-19.

To date, there is no effective drug for the treatment of COVID-19. Although different types of vaccines have been approved to be used worldwide, the number of vaccines is limited and the protection efficiency varies across countries and regions. Therefore, the severity of the COVID-19 pandemic remains intact and the number of infected people keeps increasing, especially in India. Furthermore, at least three types of SARS-CoV-2 mutants have been identified: B.1.1.7, which was first found in the United Kingdom (79); E484K, which was first found in South Africa (80); and the Indian mutant (the delta variant), which has higher transmission efficiency and stronger pathogenicity than other variants of the virus (81). Therefore, effective drugs are urgently required for the treatment of COVID-19, especially for patients with comorbidities such as hypertension, cardiovascular disease, and lung injury. Of note, several drugs are being developed to treat COVID-19, and some of them are in phase 3 of the clinical trial. For example, colchicine (phase 3) is investigated to determine whether short-term treatment with colchicine reduces the rate of death and lung complications associated with COVID-19. Convalescent plasma has been also used in an efficacious therapy to prevent the progression from mild to severe/critical COVID-19.

Although the putative effects of ACE2 downregulation on the cardiovascular system in the course of the COVID-19 pandemic requires more intensive studies, patients with COVID-19 with these comorbidities of cardiovascular disease have already been treated with RAS and ACE2 regulators. Recent research supports continued use of drugs such ARBs or ACE-Is for patients who have been already using these medications before SARS-CoV-2 infection (82). Thus, we speculate that ACE2-based regulation strategies may become one of the most promising approaches for future therapies and improve disease prognosis in COVID-19. This review will serve as a point of reference for the use of these related drugs.

## Author Contributions

YH and XL designed the content. YH and LL wrote the main manuscript. LL performed the data collection. YH, LL, and XL edited the manuscript. All authors contributed to the article and approved the submitted version.

## Conflict of Interest

The authors declare that the research was conducted in the absence of any commercial or financial relationships that could be construed as a potential conflict of interest.

## Publisher’s Note

All claims expressed in this article are solely those of the authors and do not necessarily represent those of their affiliated organizations, or those of the publisher, the editors and the reviewers. Any product that may be evaluated in this article, or claim that may be made by its manufacturer, is not guaranteed or endorsed by the publisher.
